# eCD4-Ig promotes ADCC activity of sera from HIV-1-infected patients

**DOI:** 10.1371/journal.ppat.1006786

**Published:** 2017-12-18

**Authors:** Meredith E. Davis-Gardner, Matthew R. Gardner, Barnett Alfant, Michael Farzan

**Affiliations:** Department of Immunology and Microbiology, The Scripps Research Institute, Jupiter, Florida, United States of America; University of North Carolina at Chapel Hill, UNITED STATES

## Abstract

Antibody-dependent cell-mediated cytotoxity (ADCC) can eliminate HIV-1 infected cells, and may help reduce the reservoir of latent virus in infected patients. Sera of HIV-1 positive individuals include a number of antibodies that recognize epitopes usually occluded on HIV-1 envelope glycoprotein (Env) trimers. We have recently described eCD4-Ig, a potent and exceptionally broad inhibitor of HIV-1 entry that can be used to protect rhesus macaques from multiple high-dose challenges with simian-human immunodeficiency virus AD8 (SHIV-AD8). Here we show that eCD4-Ig bearing an IgG1 Fc domain (eCD4-IgG1) can mediate efficient ADCC activity against HIV-1 isolates with differing tropisms, and that it does so at least 10-fold more efficiently than CD4-Ig, even when more CD4-Ig molecules bound cell surface-expressed Env. An ADCC-inactive IgG2 form of eCD4-Ig (eCD4-IgG2) exposes V3-loop and CD4-induced epitopes on cell-expressed trimers, and renders HIV-1-infected cells susceptible to ADCC mediated by antibodies of these classes. Moreover, eCD4-IgG2, but not IgG2 forms of the broadly neutralizing antibodies VRC01 and 10–1074, enhances the ADCC activities of serum antibodies from patients by 100-fold, and significantly enhanced killing of two latently infected T-cell lines reactivated by vorinostat or TNFα. Thus eCD4-Ig is qualitatively different from CD4-Ig or neutralizing antibodies in its ability to mediate ADCC, and it may be uniquely useful in treating HIV-1 infection or reducing the reservoir of latently infected cells.

## Author summary

Antibodies can bind HIV-1-infected cells by recognizing the viral envelope glycoprotein (Env) on the cell surface. Antibody-bound cells then recruit natural killer cells to eliminate these infected cells. Here we demonstrate the unique properties of eCD4-Ig, a potent and exceptionally broad antibody-like HIV-1 entry inhibitor. Like antibodies, eCD4-Ig can efficiently mediate killing of infected cells. However, unlike most antibodies, eCD4-Ig can promote Env conformational changes that then allow abundant but otherwise inert antibodies in patient sera to mediate killing of infected cells. This property may be especially useful in efforts to cure HIV-1 by reactivating virus in latently infected cells and then killing these virus-producing cells.

## Introduction

Natural killer (NK) cells and other Fc-gamma receptor (FcγR)-expressing cells can eliminate HIV-1 infected cells through antibody-dependent cell-mediated cytotoxicity (ADCC) [1–3]. These ADCC activities depend on the antibody isotype [4,5]. For example, IgG1 and IgG3 mediate efficient ADCC, whereas the IgG2 and IgG4 do so poorly. Growing evidence suggests that ADCC is an important component of protective immune responses against HIV-1 infection [1,2,6–8]. High levels of ADCC antibodies have been correlated with better disease prognosis and slower disease progression in HIV-infected individuals [1,3,9]. Further, analysis of the only human vaccine trial reporting some protection, RV144, identified ADCC-mediating antibodies as a correlate of protection [2,6]. Studies of passively administered antibodies in animal models have suggested that ADCC contributes to protection from simian-human immunodeficiency viruses (SHIV) [10]. The ability of some antibody-treated macaques to maintain low viral loads after cessation of treatment suggests that, in some contexts, antibodies can help eliminate infected cells and boost host immune responses so they can subsequently control viral replication [11]. Efforts are now underway to reduce the viral reservoir by combining latency-reversing agents, which stimulate virion production in latently infected cells, with antibodies that may accelerate elimination of these cells [12,13].

Engineered biologics with IgG1 Fc domains can also mediate ADCC [14]. We have developed one such inhibitor, eCD4-Ig, that is as potent as most HIV-1 broadly neutralizing antibodies (bNAbs), but which uniquely neutralizes 100% of tested HIV-1, HIV-2, and SIV isolates, all with 80% inhibitory concentrations (IC_80_s) less than 10 μg/ml [15]. eCD4-Ig is a fusion of CD4-Ig with a tyrosine-sulfated coreceptor-mimetic peptide appended to its carboxy-terminus [15,16]. The two sulfopeptides of the eCD4-Ig dimer and at least one of its CD4 domains engage Env to provide high avidity binding and prevent enhancement of infection observed with CD4-Ig. Its close emulation of the HIV-1 receptor CD4, and the HIV-1 coreceptors CCR5 and CXCR4, likely account for its exceptional breadth. The breadth and potency of eCD4-Ig has also been demonstrated *in vivo*: In macaque studies, adeno-associated virus (AAV) vector-delivered eCD4-Ig achieved complete and significant protection against multiple high-dose challenges with either SHIV-AD8 or SIVmac239 [15].

These *in vivo* studies utilized an eCD4-Ig variant with rhesus CD4 and IgG2 Fc domains, indicating that the ADCC activities of IgG1 may not be essential for prophylaxis. However, eCD4-Ig may also be useful in controlling an established infection or reducing the viral reservoir, where ADCC activities are likely more critical. We therefore investigated its ability to mediate ADCC alone and in combination with antibodies or patient sera. We observed that, despite lower occupancy of Env, eCD4-Ig mediated markedly more efficient ADCC than did CD4-Ig. Further, V3-loop and CD4-induced (CD4i) antibodies mediated more efficient ADCC in the presence of an IgG2 form of eCD4-Ig because eCD4-Ig promotes exposure of these epitopes. Most critically, eCD4-IgG2, but not bNAbs, markedly enhanced the ADCC activities of sera from six patients where weak ADCC activity was detected. Thus, unlike bNAbs, eCD4-Ig uniquely works with the host immune system to eliminate infected cells.

## Results

### eCD4-IgG1 efficiently mediates ADCC

**Fig 1:**
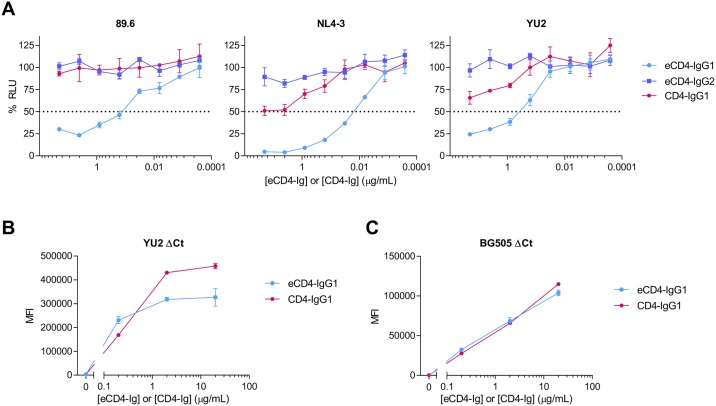
eCD4-IgG1 mediates potent ADCC activity. (**A**) CEM.NKR-CCR5-LTR-Luc cells were infected with HIV-1 isolates 89.6, NL4-3, or YU2. Three or four days post-infection, cells were mixed at a 10:1 effector to target ratio with an NK cell line expressing human CD16a in the presence of the indicated eCD4-IgG1, eCD4-IgG2, or CD4-IgG1 concentrations. ADCC responses, defined as a loss of luciferase signal in relative light units (RLU), were measured after an 8 hour incubation. Dotted line represents 50% of maximal luciferase signal from infected targets incubated with NK cells in the absence of any inhibitor. Results are expressed as means +/- standard error of mean (S.E.M.) (n = 3). Data are representative of at least three independent experiments. (**B-C**) HEK293T cells were transfected to express the YU2 (B) or BG505 (C) Env with a deletion in its cytoplasmic tail (ΔCt) to increase expression on the cell surface. Cells were then incubated with the indicated concentrations of CD4-IgG1 or eCD4-IgG1, washed, and binding was measured by flow cytometry using a secondary antibody recognizing the human Fc domain. Error bars represent a range of two measurements.

If eCD4-Ig is to be used as part of a treatment or cure regimen, its ADCC activity is likely to be important. We therefore compared the ADCC activities of CD4-Ig and eCD4-Ig with IgG1 Fc domains (CD4-IgG1, eCD4-IgG1), and eCD4-Ig with an IgG2 Fc domain (eCD4-IgG2). As we have previously reported, the presence of the carboxy-terminal sulfopeptide did not interfere with the ADCC activity of eCD4-IgG1, rather it enhanced it [15]. Specifically, target cells infected with a CCR5-using (R5) isolate (YU2), a CXCR4-using (X4) isolate (NL4-3), or a dual-tropic (R5X4) isolate (89.6) were more efficiently lysed by CD16a+ NK cells when incubated with eCD4-IgG1 than with CD4-IgG1 (Fig 1A). Expectedly, eCD4-IgG2 did not efficiently mediate ADCC. The greater ADCC activity of eCD4-IgG1 relative to CD4-IgG1 is somewhat surprising, because cells expressing YU2 or BG505 Env bound fewer or similar numbers of eCD4-Ig relative to CD4-Ig (Fig 1B and 1C). These observations suggest that eCD4-IgG1 presents its Fc domains in an orientation more favorable to ADCC than does CD4-IgG1.

### eCD4-Ig exposes additional Env epitopes

**Fig 2:**
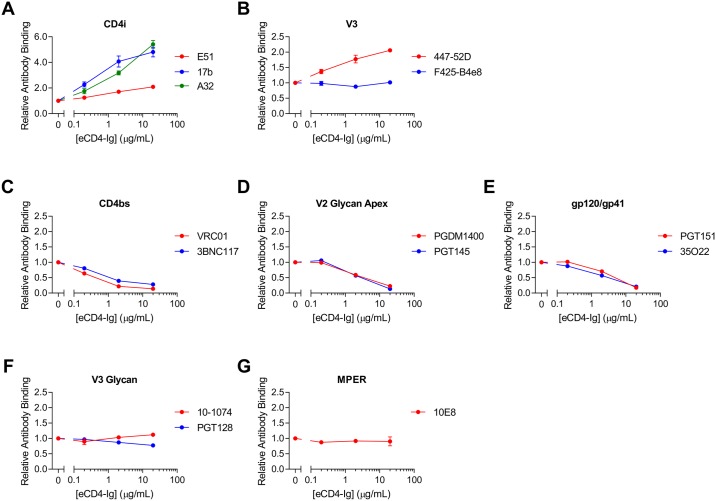
eCD4-Ig induces changes in antibody binding to HIV envelope glycoprotein. (**A-G**) HEK293T cells were transfected to express the BG505 Env with a deletion in its cytoplasmic tail to increase expression on the cell surface. Cells were pre-incubated with varying concentrations of eCD4-Ig with mouse Fc domains (eCD4-mIg), as indicated. Cells were washed and then incubated with 0.4 μg/mL of the indicated antibodies, and analyzed by flow cytometry. Mean fluorescence intensity values (MFI) are normalized to the value of antibody binding in the absence of eCD4-Ig or CD4-Ig. Error bars represent range (n = 2). Data are representative of at least three independent experiments.

eCD4-Ig binds both the receptor- and coreceptor-binding sites on HIV Env [15], and induces conformational changes that may affect access to antibody epitopes. We first investigated the ability of eCD4-Ig bearing a murine Fc domain (eCD4-mIg) to alter binding of several classes of HIV-1 neutralizing antibodies. The murine Fc domain allowed us to distinguish between binding of eCD4-Ig and HIV-1 antibodies with human Fc domains. HEK293T cells expressing BG505 Env lacking the cytoplasmic tail (BG505ΔCt) were pre-incubated with eCD4-mIg and then the resulting complexes were analyzed by flow cytometry for their ability to bind human antibodies. We observed that eCD4-mIg markedly increased binding of the CD4i antibodies 17b, E51, and A32 despite the presence of a potentially competing sulfopeptide (Fig 2A). However, as expected, CD4-Ig did so more efficiently (S1A Fig). Similarly, the epitope of the V3-loop antibody 447-52D was exposed by both CD4-mIg and eCD4-mIg, whereas the nearby epitope of the antibody F425-B4e8 was not further exposed (Fig 2B and S1B Fig). eCD4-mIg prevented binding of CD4-binding site antibodies (VRC01, 3BN117), V2 glycan apex antibodies (PGDM1400, PGT145), and antibodies recognizing the interface of Env subunits gp120 and gp41 (PGT151, 35O22) (Fig 2C–2E). In contrast, it did not affect binding of the V3-glycan antibodies (10–1074, PGT128) or an MPER class antibody (10E8) (Fig 2F and 2G) [17]. Our data suggest that eCD4-Ig might work synergistically with CD4i and V3-loop antibodies, but may antagonize CD4-binding site, apex, and interface antibodies.

### eCD4-Ig enhances the ADCC activities of V3-loop and CD4i antibodies

**Fig 3:**
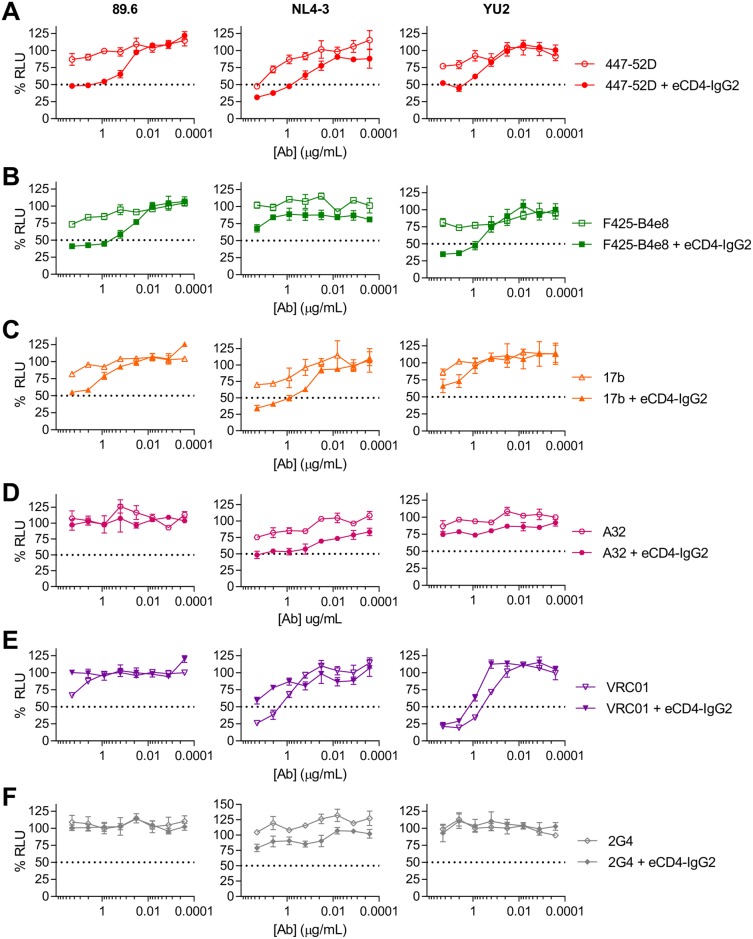
eCD4-IgG2 enhances ADCC activity of V3-loop and CD4i antibodies. An ADCC assay similar to that described in Fig 1 was used. Effector and target cells infected with HIV-1 isolates 89.6, NL4-3, or YU2 were incubated with the V3-loop antibodies 447-52D (**A**) and F425-B4e8 (**B**), the CD4i antibodies 17b (**C**) and A32 (**D**), the CD4bs antibody VRC01 (**E**), or 2G4, a control anti-Ebola glycoprotein antibody (**F**), at the indicated dilutions, either alone (open symbols) or in the presence of 1 μg/mL eCD4-IgG2 (filled symbols). ADCC activity was determined by luciferase activity after 8 hour incubation. Results are represented as means +/- S.E.M. (n = 3). Data are representative of at least three independent experiments.

The ability of eCD4-Ig to expose the epitopes of V3-loop and CD4i antibodies is of interest because these antibodies are abundant but generally non-neutralizing in infected individuals [18,19]. We therefore investigated whether eCD4-IgG2, which does not mediate ADCC, could promote the ADCC activities of antibodies from these classes. We used ADCC-inactive eCD4-IgG2 so that we could monitor the effects of eCD4-Ig on ADCC mediated solely by the HIV-1 antibodies. eCD4-IgG2 markedly enhanced ADCC mediated by the V3-loop antibodies 447-52D (Fig 3A) and F425-B4e8 (Fig 3B) in cells infected with the HIV-1 isolates 89.6 and YU2, and to a lesser extent with NL4-3. It also promoted ADCC activity of the CD4i antibodies 17b (Fig 3C) and A32 (Fig 3D), whereas it attenuated ADCC mediated by the CD4-binding site antibody VRC01 (Fig 3E). eCD4-IgG2 did not alter ADCC mediated by 2G4, a control antibody recognizing Ebola virus GP_1,2_ (Fig 3F). We also analyzed the ability of these antibodies to neutralize the same viruses (S2 Fig). Notably, eCD4-IgG2 could enhance the ADCC activity of antibodies that were non-neutralizing. For example, neither antibody recognizing the V3-loop of Env (447-52D and F425-B4e8) neutralized the YU2 isolate, but in the presence of eCD4-IgG2, both could mediate ADCC. Thus eCD4-IgG2, which does not itself mediate ADCC, nonetheless can enhance the ADCC activity of non-neutralizing antibodies.

### eCD4-Ig promotes ADCC activity of serum from HIV-1-infected patients

**Fig 4:**
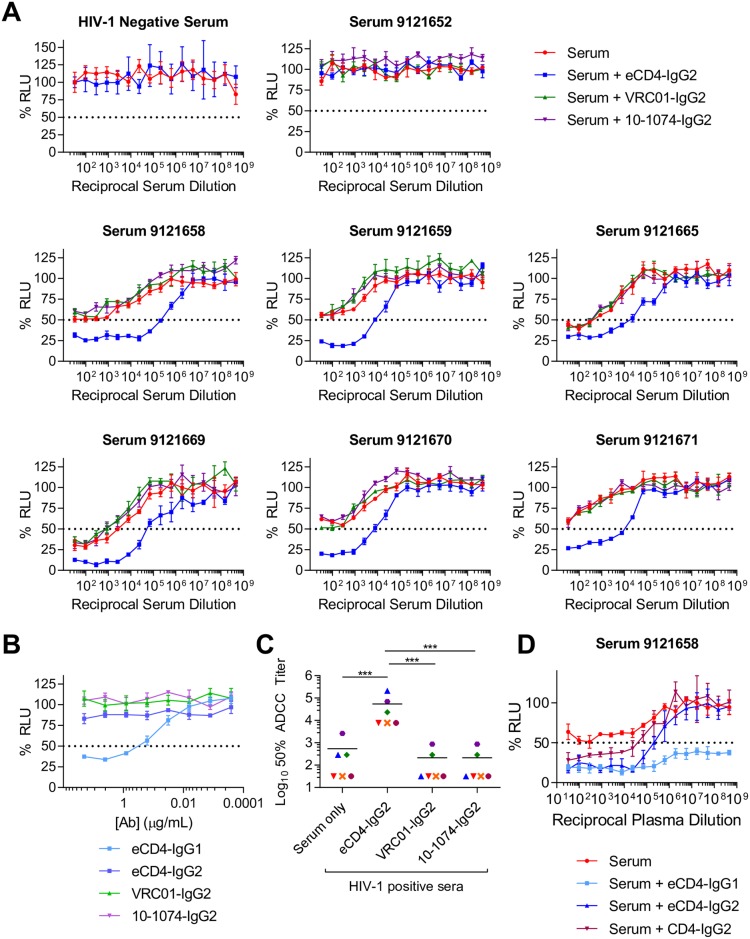
eCD4-IgG2 enhances ADCC activity of HIV-1 infected sera. **(A)** ADCC assays similar to those described in Fig 1. CEM.NKR-CCR5-LTR-Luc target cells were infected for 3 days with YU2. Effector cells were added at a 10:1 ratio in the presence of 3-fold serial dilutions beginning at 1:32 dilution of human sera from uninfected or HIV-1 positive patients alone (red) or in the presence of 1 μg/mL eCD4-IgG2 (blue), VRC01-IgG2 (green), or 10-1074-IgG2 (purple). ADCC activity was determined by luciferase activity after an 8 hour incubation. **(B)** ADCC activity of eCD4-IgG1, eCD4-IgG2, VRC01-IgG2, and 10-1074-IgG2 was measured in a similar manner as in (A). Results are represented as means +/- S.E.M. (n = 3). Data are representative of at least two independent experiments. **(C)** 50% ADCC titers were calculated from curves in (A) as the dilution at which lines crossed the 50% maximal luciferase value. Paired t-test, ***p<0.001. (**D**) ADCC assay similar to those described in A. In this case, ADCC activity of serum 9121658 was tested alone (red) and in combination with 1 μg/mL eCD4-IgG1 (light blue), eCD4-IgG2 (dark blue), or CD4-IgG2 (burgundy).

The ability of eCD4-IgG2 to enhance the ADCC activity of V3-loop and CD4i antibodies raises the possibility that it may similarly promote ADCC in serum from HIV-1-infected patients, where these antibodies are abundant. To test this possibility, we initially screened de-identified sera from 15 patients for their ability to mediate ADCC against the YU2 isolate, and to neutralize the same isolate. Six of these sera mediated ADCC against YU2 but only one efficiently neutralized the same virus, with 50% neutralization at a 1:824 dilution (S3 Fig). We further evaluated ADCC activity of the six ADCC-active sera, as well as one ADCC-inactive serum (9121652) and serum from an HIV-1-negative individual. ADCC activity was measured in the presence or absence of eCD4-IgG2, or IgG2 forms of the bNAbs VRC01 and 10–1074 (Fig 4A). In all six cases, eCD4-IgG2 markedly promoted the ADCC activity of these patient sera, typically by 100-fold. eCD4-IgG2 did not alter the ADCC activity of HIV-1-negative serum or of HIV-1-positive but ADCC-inactive serum. In contrast, IgG2 forms of VRC01 and 10–1074 which, like eCD4-IgG2, do not mediate ADCC (Fig 4B), had no effect on the ADCC activity of patient sera. ADCC activity in the presence of eCD4-IgG2 was significantly greater than with sera alone (p = 0.0001), or in the presence of VRC01 or 10–1074 (both p = 0.0001; Fig 4C). Thus eCD4-IgG2, but not IgG2 forms of broadly neutralizing antibodies, can dramatically enhance the ADCC activities of sera from infected individuals. We additionally tested the effect of serum in combination with eCD4-IgG1 or CD4-IgG2 (Fig 4D). As expected, CD4-IgG2 synergized with serum, but not as efficiently as eCD4-IgG2. Also expectedly, eCD4-IgG1 combined with serum to afford highly potent ADCC activity, likely a combination of potent ADCC-activity of 1 μg/ml of eCD4-IgG1 itself (see Fig 4B) and its ability to synergize with patient serum. We conclude that patient serum is likely to improve the already potent ADCC activity of eCD4-IgG1. Collectively the data of Figs 2–4 show that, among potent HIV-1 entry inhibitors, eCD4-Ig can uniquely unmask epitopes of antibodies present in the sera of HIV-1-infected patients, likely because it alters the conformation of Env on the cell surface.

### eCD4-Ig promotes ADCC activity against reactivated latently infected cells

**Fig 5:**
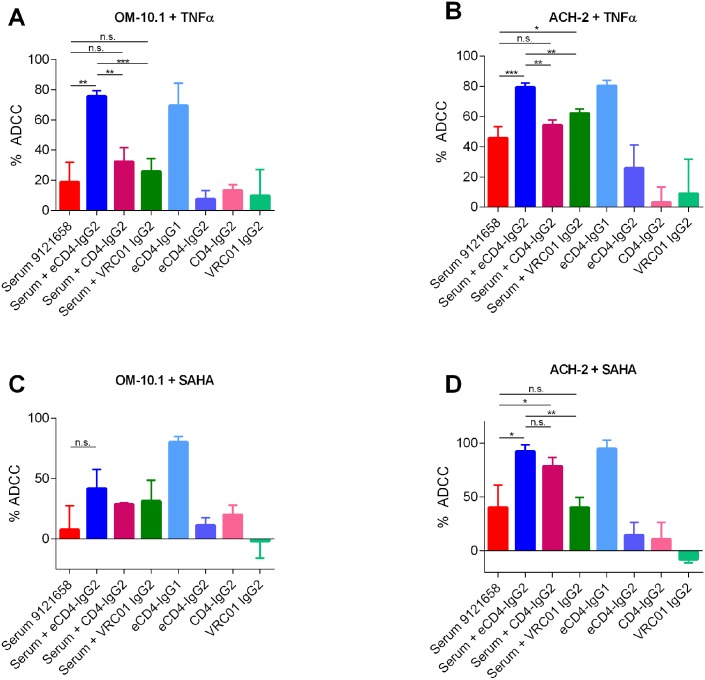
eCD4-IgG2 enhances ADCC activity of HIV-1 infected sera against reactivated latently infected cells. OM-10.1 (**A**, **C**) or ACH-2 (**B**, **D**) cells lines were reactivated with TNFα (**A**, **B**) or vorinostat (SAHA) (**C**, **D**) for 24 hours prior to being mixed at a 10:1 effector to target ratio with an NK cell line expressing human CD16a in the presence of a 1:1000 dilution of serum 9121658 alone or in combination with 1 μg/mL of eCD4-IgG2, or CD4-IgG2, or VRC01-IgG2. ADCC responses, calculated based on intracellular p24 staining, were measured after a 5 hour incubation. Results are expressed as means +/- S.E.M. (n = 3). Data are representative of at least three independent experiments. Unpaired t-test, ***p<0.001, **p<0.01, *p<0.05, n.s. not significant.

One potential application of eCD4-Ig as a therapeutic would be as part of a “shock and kill” approach to a sterilizing cure. This approach seeks to eliminate the latent viral reservoir by reactivating latent provirus using a latency reversing agent (LRA) in combination with an agent that accelerates killing of reactivated cells. eCD4-Ig might be uniquely useful in this regard as both the IgG1 and IgG2 forms can neutralize newly produced virions as well as target reactivated cells for killing by ADCC, either directly by eCD4-IgG1 or by sensitizing cell-surface expressed Env to other circulating antibodies. To assess the potential of eCD4-Ig in this context, we investigated its ability to direct killing of reactivated latently infected cell lines (Fig 5). OM-10.1 (Fig 5A and 5C) or ACH-2 (Fig 5B and 5D) cells were reactivated using either the cytokine TNFα or the HDAC inhibitor, vorinostat (suberoylanilide hydroxamic acid, SAHA) and killing was measured using a flow cytometry-based ADCC assay. In all cases, eCD4-IgG1 showed potent ADCC activity. Additionally, while HIV+ serum 9121658 had some ADCC activity alone, ADCC activity was increased by the addition of 1 μg/mL eCD4-IgG2. Increases in these activities were significant when both cell types were reactivated with TNFα, and when ACH-2 cells were reactivated with vorinostat. A similar trend was observed with OM-10.1 cells reactivated with vorinostat, but these changes were not significant. As we previously observed in the luciferase-based assay (Fig 4D), eCD4-IgG2 was more potent at promoting serum ADCC activity than was CD4-IgG2 and this difference was significant in both cell lines reactivated with TNFα. These results suggest that eCD4-Ig may make an especially potent ‘kill’ for a shock and kill approach to eliminating the reservoir of latently infected cells.

## Discussion

eCD4-Ig is a potent and exceptionally broad inhibitor of HIV-1 entry [15]. Here we examined its ability to mediate ADCC in several contexts. We first compared it with CD4-Ig, using an R5, an X4, and an R5X4 isolate. In each case, eCD4-IgG1 more efficiently mediated cell killing relative to CD4-IgG1 (Fig 1A). This enhanced ADCC activity is consistent with its higher avidity binding to Env, but still somewhat surprising because fewer eCD4-Ig than CD4- molecules bound the Env trimer (Fig 1B). These observations suggest that eCD4-Ig binds Env in a different and perhaps more stable orientation than does CD4-Ig, likely because sulfopeptide-binding fixes the orientation of the Fc domain. Alternatively, eCD4-Ig may bridge two or more Env trimers, perhaps more efficiently crosslinking FcγRIIIa on NK cells. In either case, the manner in which eCD4-Ig mediates ADCC appears to be qualitatively different from CD4-Ig.

We also identified an important difference between eCD4-Ig and broadly neutralizing antibodies. Specifically, eCD4-Ig, like soluble CD4 and less-potent CD4-mimetic compounds [20–24], induces conformational changes in Env (Fig 2) that promote ADCC mediated by V3-loop and CD4i antibodies (Fig 3). These antibodies are frequently found in HIV-1-positive individuals [18,19], although by themselves they do little to control an infection. Most importantly, we show that, in contrast to antibodies, eCD4-IgG2, an ADCC-inactive form of eCD4-Ig, increased the ADCC activity of serum from infected patients much more effectively than any previously described agent (Figs 4D and 5), presumably by enlisting the help of antibodies that would not otherwise bind Env. Of note, eCD4-IgG2 did so more effectively than CD4-IgG2 (Figs 4D and 5), perhaps because the coreceptor-mimetic sulfopeptide contributes to the stability of Env in its CD4-bound conformation. The ability of eCD4-Ig to synergize with serum antibodies is potentially important if eCD4-Ig is used to control an established HIV-1 infection. Our data further suggest that, despite its lack of direct effector functions, eCD4-IgG2 can mediate these activities should the safety of eCD4-IgG1 be a concern. However, as we show in Fig 4D, ADCC-active eCD4-IgG1 can similarly combine with these antibodies to mediate even more robust killing of infected cells. Indeed, given its exceptional breadth, its potent intrinsic ADCC activity, and its ability to synergize with otherwise weakly active endogenous antibodies to further mediate ADCC, eCD4-IgG1 is likely to be more effective *in vivo* than any bNAb at eliminating infected cells. It is also possible that therapeutic vaccines that actively raise V3-loop and CD4i antibodies, perhaps by locking soluble Env trimers in the CD4-bound state [25], may further improve eCD4-IgG1-mediated cell killing.

One context in which the unique ADCC properties of eCD4-Ig might be especially useful is in efforts to reduce or eliminate the reservoir of latently infected cells in a “shock and kill” approach to cure HIV-1 [12,13]. This approach relies on an agent such as a histone deacetylase inhibitor, for example vorinostat, or a TLR7 agonist to reactivate the latent HIV-1 provirus in infected cells, which can then be eliminated through ADCC or other mechanisms. The properties of eCD4-Ig shown here suggest that it might be more effective for this purpose than other CD4-mimetic compounds or bNAbs. Specifically, we show that eCD4-IgG2 can synergize with patient serum to kill cells reactivated with either vorinostat or TNFα, as can eCD4-IgG1 alone (Fig 5). Unlike small CD4-mimetic peptides and small molecules, eCD4-Ig can also potently neutralize virus, its IgG1 form can mediate ADCC directly, and both IgG1 and IgG2 forms can boost the ADCC activities of patient sera more effectively than any other agent. eCD4-Ig is likely more efficient and consistent at inducing the CD4-bound conformation than these small compounds, likely accounting for the inability of at least one such compound to promote serum-mediated killing of reactivated ACH-2 cells [24]. eCD4-Ig has key advantages over bNAbs as part of a shock and kill strategy. In general bNAbs do not promote the ADCC activities of patient sera, and their more limited breadth would preclude killing of every reactivated cell.

In summary, we have shown that eCD4-Ig is more effective than CD4-Ig at mediating ADCC, and that, unlike broadly neutralizing antibodies, it can dramatically enhance weak ADCC activity of sera from infected patients. These properties may be useful in efforts to eliminate the viral reservoir.

## Materials and methods

### Cells and plasmids

HEK293T (ATCC, Manassas, VA) and TZM-bl cell lines were grown in DMEM supplemented with 10% fetal bovine serum. TZM-bl cells were obtained through the NIH AIDS Reagent Program, Division of AIDS, NIAID, NIH, contributed by Dr. John C. Kappes, Dr. Xiayun Wu and Tranzyme Inc [26–30]. Expi293F cells were grown in Expi293 Expression media (Life Technologies, Carlsbad, CA).

ADCC target and effector cells have been described previously and were a generous gift from Drs. Michael Alpert and David Evans. Briefly, CEM.NKR-CCR5-LTR-Luc ADCC target cells, harboring an HIV-1 Tat-inducible luciferase, were derived from CEM.NKR-CCR5 CD4^+^ T Cells obtained from the NIH AIDS Research and Reference Reagent Program (ARRRP), Division of AIDS, NIAID, NIH, contributed by Dr. Alexandra Trkola and have been previously described [31–35]. Targets cells were grown in R10 media, specifically RPMI supplemented with 10% FBS, 25 mM HEPES, 2 mM L-glutamine, and 0.1 mg/mL Primocin (InvivoGen, San Diego, CA). KHYG-1 derived NK cell line expressing human CD16a (V158 variant) have been previously described [31,32]. NK cells were grown in R10 media additionally supplemented with 1 μg/mL cyclosporine, and interleukin-2 (IL-2). OM-10.1 and ACH-2 cells were obtained from the NIH AIDS reagent Program, Division of AIDS, NIAID, NIH: OM-10.1 Cells from Dr. Salvatore Butera [36–40], ACH-2 from Dr. Thomas Folks [41,42]. Both OM-10.1 and ACH-2 cells were grown in RPMI supplemented with 10% FBS, 25 mM HEPES, 2 mM L-glutamine, 100 U/ml penicillin, 100 μg/ml streptomycin.

The variable heavy and light chains of 447-52D, F425-B4e8, 17b, E51, A32, PGDM1400, PGT145, PGT151, and 2G4 were cloned into human IgG1 expression vectors as previously described [43]. Vectors expressing VRC01 heavy and light chains were obtained through the NIH AIDS Reagent Program, Division of AIDS, NIAID, NIH, from Dr. John Mascola [44,45]. 3BNC117 and 10–1074 IgG1 expression plasmids were provided by Dr. Michel Nussensweig. 10E8 expression vectors were obtained through the NIH AIDS Reagent Program, Division of AIDS, NIAID, NIH, from Dr. Mark Connors [46]. PGT121, PGT128 were provided by Dr. Dennis Burton. 35O22 expression vectors were obtained through the NIH AIDS Reagent Program, Division of AIDS, NIAID, NIH: Cat# 12584 mAb 35O22 heavy chain expression vector (CMVR) and Cat# 12585 mAb 35O22 light chain, from Drs. Jinghe Huang and Mark Connors [47]. Heavy and light chains for 447-52D and F425-B4e8 were synthesized by Integrated DNA Technologies (IDT, Newark, NJ) and cloned into IgG1 expression vectors. Plasmids encoding IgG2 forms of VRC01 and 10–1074 were generated by replacing genes encoding human IgG1 constant regions with those of human IgG2. Expression vectors for the BG505 gp160-Δcytoplasmic tail were provided by Drs. John Moore and PJ Klasse. eCD4-IgG1 has been previously described [15,16]. Briefly, eCD4-Ig is an Fc-fusion protein of CD4 domains 1 and 2 with the addition of a sulfated CCR5-mimetic peptide at the C-terminus. A plasmid encoding eCD4-IgG2 was generated by replacing sequence encoding the human IgG1 Fc domain in the eCD4-IgG1 expression plasmid with that of IgG2.

### Antibody and eCD4-Ig production and purification

Antibodies and eCD4-Ig were produced in Expi293 cells (Life Technologies, Carlsbad, CA). Cells were grown to a density of 3x10^6^ cells/mL prior to transfection with Expifectamine according to manufacturer’s instructions (Life Technologies, Carlsbad, CA). 140 μg total DNA was transfected in 250 mL Expi293 cells. eCD4-IgG1 and eCD4-IgG2 plasmids were cotransfected at an 4:1 ratio with plasmid encoding human tyrosine protein sulfotransferase 2 (TPST2). Antibodies were produced by transfection of two plasmids encoding heavy and light chain, respectively, at a 1:1 ratio. 20 hours post-transfection, Expifectamine enhancers were added according to manufacturer’s instructions. 5 days post-transfection, media was collected for protein purification. Debris was cleared by centrifugation for 10 min at 4000g and filtered using 0.45-μm filter flasks (Thermo Scientific, Waltham, MA). Antibodies and Fc-fusion proteins were purified from supernatants using HiTrap MabSelect SuRe columns (GE Healthcare Life Sciences, Pittsburgh, PA). After protein binding, columns were washed extensively with PBS before elution with IgG Elution Buffer (Thermo Scientific Waltham, MA). Eluate pH was immediately adjusted with Tris-HCl 1M pH 9.0 Neutralization Buffer (G-Biosciences, Saint Louis, MO). Buffer was exchanged with PBS and protein was concentrated to 1 mg/mL by Ultrafiltration (Amicon Ultra, Millipore Sigma, Billerica, MA) at 4000 g.

### Ethics statement

De-identified HIV-1 positive serum was obtained from Boston Biomedical Inc. (BBI, Boston, MA), and has been previously described [48]. De-identified uninfected serum was purchased from Sigma (Saint Louis, MO). All materials were handled in accordance with the regulations set forth by the Scripps Office for the Protection of Research Subjects.

### Viruses

Viruses were produced in HEK293T cells following transfection of p89.6, pNL4-3, and pYU-2 HIV-1 molecular clones using calcium phosphate. Supernatants were harvested 48 hours post-transfection, filtered through 0.45-μm filters, aliquoted and frozen at -80°C. p89.6 molecular clone was obtained from the ARRRP, Division of AIDS, NIAID, NIH, deposited by Dr. Ronald G. Collman, MD [49–51]. pNL4-3 molecular clone was obtained from the ARRRP, Division of AIDS, NIAID, NIH, from material deposited by Drs. Suzanne Gartner, Mikulas Popovic, Robert C. Gallo, and Malcom Martin [52]. The pYU-2 HIV-1 molecular clone was obtained through the NIH AIDS Reagent Program, Division of AIDS, NIAID, NIH, from Dr. Beatrice Hahn and Dr. George Shaw [53,54]. Viruses titers were quantified by p24 ELISA (Advanced Bioscience Laboratories, Rockville, MD).

### Luciferase-based Antibody Dependent Cell Cytotoxicity assay

ADCC assay was performed as previously described [31,32]. Briefly, CEM.NKR-CCR5-LTR-Luc target cells were infected by spinoculation with 89.6 (400ng p24) and NL4-3 (200ng p24) 4 days and YU-2 (500ng p24) 3 days prior to assay. Infection amounts for each assay were determined by virus titration on the target cells. On the day of the assay, infected target cells were mixed with NK effector cells at a 10:1 ratio in the presence of antibodies or eCD4-Ig. Cell and inhibitor mixes were incubated for 8 hours at 37°C. ADCC activity was measured by luciferase using BriteLite Plus (Perkin Elmer, Waltham, MA) and measured using a Victor X3 plate reader (Perkin Elmer, Waltham, MA).

### Flow cytometry-based Antibody Dependent Cell Cytotoxicity assay

ADCC against latently infected OM-10.1 and ACH-2 cells was measured using a flow cytometry-based assay. Specifically, cells were reactivated with 10 ng/mL TNFα (Life Technologies, Carlsbad, CA) or 1 μM SAHA (Sigma-Aldrich, Saint Louis, MO) for 24 hours prior to assay. Reactivated cells were incubated with NK-V158 cells at a 10:1 ratio in the presence of eCD4-Ig, CD4-Ig, or antibodies at a concentration of 1 μg/mL or HIV-1+ serum at a 1:1000 dilution. Target cells were fixed and permeabilized using the Fix and Perm kit (Life Technologies, Carlsbad, CA) and stained for intracellular p24 expression using FITC-conjugated clone KC57 (Beckman Coulter, Brea, CA). Data were collected using Accuri C6 Flow Cytometer and data analyzed with the C6 Software (BD Biosciences, San Jose, CA). Percent ADCC was normalized to infected cells in the presence of effectors but no inhibitor, and calculated with the following formula: [(%p24+ cells in targets + effectors − inhibitor) − (%p24+ cells in targets + effectors + inhibitor)]/(%p24+ cells in targets + effectors − inhibitor) × 100.

### TZM-bl neutralization assay

TZM-bl neutralization assays were performed as previously described [15,16,43]. Briefly, eCD4-Ig or antibody titrations were incubated with infectious viruses for 1 hour at 37°C. TZM-bl cells were diluted in DMEM to 100,000 cells/mL and added to the virus/inhibitor mix. Cells were then incubated for 40 hours at 37°C. Viral entry was determined by luciferase readout with BriteLite Plus (Perkin Elmer, Waltham, MA) and read on a Victor X3 plate reader (Perkin Elmer, Waltham, MA).

### HIV Env surface staining assay

HEK293T cells were transfected with plasmids expressing HIV-1 envelope glycoprotein variants lacking cytoplasmic residues 732–876 (HXBc2 numbering). Cells were collected 48 hours post transfection with non-enzymatic dissociation buffer (Sigma-Aldrich, Saint Louis, MO). Cells were washed with flow cytometry buffer (PBS with 2% goat serum, 0.01% sodium azide) before incubation with eCD4-mouse-Ig for 1 hour on ice. Cells were subsequently incubated with human IgG1-containing antibodies. Antibody binding was determined with FITC-conjugated goat anti-mouse, and APC-conjugated goat anti-human secondary antibodies (Jackson ImmunoResearch, West Grove, PA). Between each antibody incubation, cells were washed twice with flow cytometry buffer. After incubation with secondary antibody, cells were washed once with flow cytometry buffer, once with PBS, and then resuspended in 1% paraformaldehyde in PBS. Binding was analyzed with an Accuri C6 Flow Cytometer and data analyzed with the C6 Software (BD Biosciences, San Jose, CA).

## Supporting information

S1 Fig Differences in induction of V3 and CD4i antibody binding between eCD4-Ig and CD4-Ig. (**A-B**) HEK293T cells were transfected to express the BG505 Env with a deletion in its cytoplasmic tail to increase expression on the cell surface. Cells were pre-incubated with varying concentrations of eCD4-Ig (solid lines) or CD4-Ig (dotted lines) with mouse Fc domains (eCD4-mIg, CD4-mIg), as indicated. Cells were washed and then incubated with 0.4 μg/mL of the indicated antibodies, and analyzed by flow cytometry. Mean fluorescence intensity values (MFI) are normalized to the value of antibody binding in the absence of eCD4-Ig or CD4-Ig. Error bars represent range (n = 2). Data are representative of at least three independent experiments.
(TIF)

S2 Fig *In vitro* neutralization by eCD4-Ig and monoclonal antibodies. HIV-1 isolates 89.6, NL4-3, or YU2 were incubated for 1 hour with 5-fold serial dilutions of eCD4-IgG1 or IgG2 or the indicated antibodies. TZM-bl cells were then added to virus-inhibitor combinations and incubated for 40 hours. Infection is represented as the percentage of luciferase activity in the absence of inhibitor. Values represent means +/- S.E.M. (n = 3). Data are representative of at least three independent experiments.
(TIF)

S3 Fig ADCC and neutralization activity screen of HIV-1 infected human sera. (**A**) ADCC assays similar to those described in Fig 1. CEM.NKR-CCR5-LTR-Luc target cells were infected for 3 days with the HIV-1 isolate YU2. Effector cells were added at a 10:1 ratio in the presence of 5-fold serial dilutions of human sera beginning at a total serum dilution of 1:32. ADCC activity was determined by luciferase activity after an 8 hour incubation. Colored symbols represent ADCC-active sera used the subsequent experiments. Grey indicates ADCC-inactive sera. (**B**) An *in vitro* neutralization assay performed as in S2 Fig. YU2 was incubated for 1 hour with 3-fold serial dilutions of indicated sera, beginning at a 1:60 dilution. TZM-bl cells were added to virus-sera combinations and incubated for 40 h. Infection is represented as the percentage of luciferase activity in the absence of inhibitor. Values represent mean +/- S.E.M. (n = 3). Data are representative of at least two independent experiments.
(TIF)
