# Supplementary figures and images for: eCD4-Ig promotes ADCC activity of sera from HIV-1-infected patients
Source: PLoS Pathog. 2017 Dec 18;13(12):e1006786. doi: 10.1371/journal.ppat.1006786 (PMC5749896; doi:10.1371/journal.ppat.1006786)

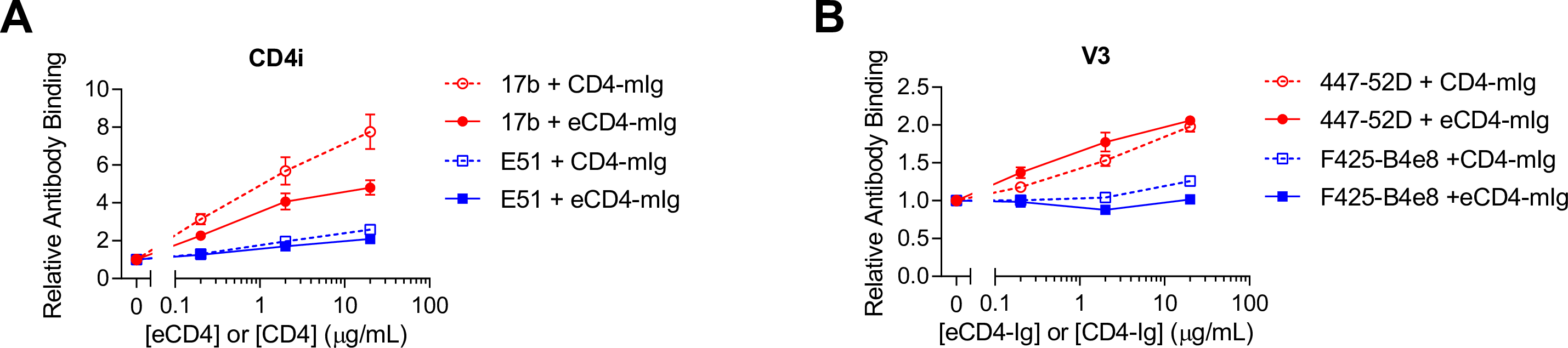

Supplement: S1 Fig — (A-B) HEK293T cells were transfected to express the BG505 Env with a deletion in its cytoplasmic tail to increase expression on the cell surface. Cells were pre-incubated with varying concentrations of eCD4-Ig (solid lines) or CD4-Ig (dotted lines) with mouse Fc domains (eCD4-mIg, CD4-mIg), as indicated. Cells were washed and then incubated with 0.4 μg/mL of the indicated antibodies, and analyzed by flow cytometry. Mean fluorescence intensity values (MFI) are normalized to the value of antibody binding in the absence of eCD4-Ig or CD4-Ig. Error bars represent range (n = 2). Data are representative of at least three independent experiments. (TIF) [file ppat.1006786.s001.tif]

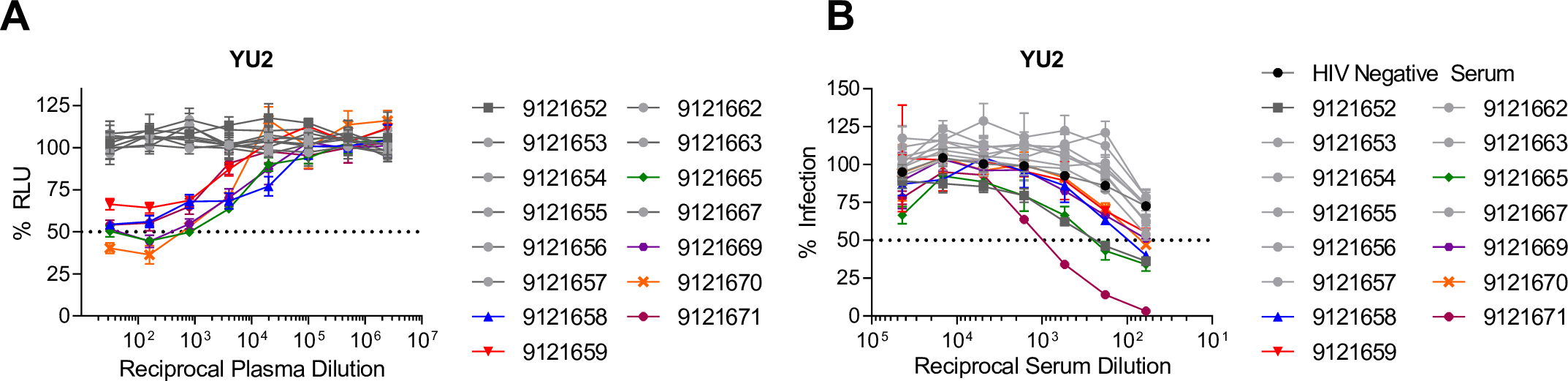

Supplement: S3 Fig — (A) ADCC assays similar to those described in Fig 1. CEM.NKR-CCR5-LTR-Luc target cells were infected for 3 days with the HIV-1 isolate YU2. Effector cells were added at a 10:1 ratio in the presence of 5-fold serial dilutions of human sera beginning at a total serum dilution of 1:32. ADCC activity was determined by luciferase activity after an 8 hour incubation. Colored symbols represent ADCC-active sera used the subsequent experiments. Grey indicates ADCC-inactive sera. (B) An in vitro neutralization assay performed as in S2 Fig. YU2 was incubated for 1 hour with 3-fold serial dilutions of indicated sera, beginning at a 1:60 dilution. TZM-bl cells were added to virus-sera combinations and incubated for 40 h. Infection is represented as the percentage of luciferase activity in the absence of inhibitor. Values represent mean +/- S.E.M. (n = 3). Data are representative of at least two independent experiments. (TIF) [file ppat.1006786.s003.tif]
